# The role of the ventrolateral anterior temporal lobes in social cognition

**DOI:** 10.1002/hbm.25976

**Published:** 2022-06-18

**Authors:** Eva Balgova, Veronica Diveica, Jon Walbrin, Richard J. Binney

**Affiliations:** ^1^ School of Human and Behavioural Sciences Bangor University Gwynedd Wales UK; ^2^ Faculdade de Psicologia e de Ciências da Educação Universidade de Coimbra Portugal

**Keywords:** anterior temporal lobe, distortion‐corrected fMRI, semantic memory, social cognition, theory of mind

## Abstract

A key challenge for neurobiological models of social cognition is to elucidate whether brain regions are specialised for that domain. In recent years, discussion surrounding the role of anterior temporal regions epitomises such debates; some argue the anterior temporal lobe (ATL) is part of a domain‐specific network for social processing, while others claim it comprises a domain‐general hub for semantic representation. In the present study, we used ATL‐optimised fMRI to map the contribution of different ATL structures to a variety of paradigms frequently used to probe a crucial social ability, namely ‘theory of mind’ (ToM). Using multiple tasks enables a clearer attribution of activation to ToM as opposed to idiosyncratic features of stimuli. Further, we directly explored whether these same structures are also activated by a non‐social task probing semantic representations. We revealed that common to all of the tasks was activation of a key ventrolateral ATL region that is often invisible to standard fMRI. This constitutes novel evidence in support of the view that the ventrolateral ATL contributes to social cognition via a domain‐general role in semantic processing and against claims of a specialised social function.

## Graphical abstract

A key challenge for neurobiological models of social cognition is to elucidate whether brain regions are specialised for that domain. We used ATL‐optimised fMRI to map the contribution of different ATL structures to a variety of paradigms frequently used to probe ‘theory of mind’, as well as a non‐social task probing semantic representations. We provide novel evidence in support of the view that the ventrolateral ATL contributes to social cognition via a domain‐general role in semantic processing and against claims of a specialised social function.

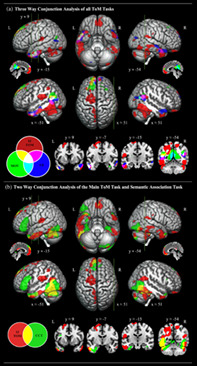

## 1 INTRODUCTION

The anterior temporal lobe (ATL) plays a crucial role in support of social cognition (Binney & Ramsey, 2020; Frith & Frith, 2003, 2010; Olson et al., 2013). Damage to the ATL results in profound and wide‐ranging socio‐affective deficits in both primates and humans (Binney, Henry, et al., 2016; Edwards‐Lee et al., 1997; Irish et al., 2014; Klüver & Bucy, 1937; Kumfor et al., 2013; Kumfor, Hazelton, et al., 2017; Kumfor, Honan, et al., 2017; Kumfor & Piguet, 2012; Terzian & Dalle Ore, 1955). Amongst neurotypical samples, the findings of functional neuroimaging studies suggest an almost ubiquitous involvement in the high‐level processing of faces and emotions (Avidan et al., 2014; Collins et al., 2016; Collins & Olson, 2014; Ramot et al., 2019; Wong & Gallate, 2012), as well as in more abstracted forms of social processing, such as moral cognition and mental state attribution (also known as theory of mind [ToM]) (Diveica et al., 2021; Molenberghs et al., 2016; Moll et al., 2005; Schurz et al., 2014).

Despite this, across various neurocognitive frameworks of the ‘social brain’, there is no firm consensus regarding the nature of the function that the ATL performs (for a comprehensive review, see Olson et al., 2007, 2013). There are likely two main drivers for this. First, at a glance, it might be difficult to identify a common cognitive process that connects the various social and emotional tasks that implicate the ATL (Binney & Ramsey, 2020; Olson et al., 2013). Second, inconsistent anatomical definitions of the ATL have made it difficult for researchers to agree where purported function is localised and where it differentiates. From one perspective, the term ‘ATL’ refers to all cortex comprising the anterior half of the temporal lobe (Binney et al., 2010; Binney, Hoffman et al., 2016; Rice et al., 2018; Rice, Hoffman, et al., 2015), and therefore a large area potentially comprising a number of functionally distinct subregions. However, it has at times been used to more specifically refer to the temporal polar cortex and the limited boundaries of Brodmann's area 38 (e.g., Ross & Olson, 2010; Simmons et al., 2010). Therefore, the broad aim of the present study was to contribute to a more complete description of the ATL subregions engaged in service of social cognitive tasks and advance understanding of the nature of their function. However, our primary aim was to use fMRI to specifically evaluate a potential contribution of the ventrolateral ATL and for two reasons. First, the ventrolateral ATL (along with the temporal pole) is the site of maximal damage in a neurological condition, known as semantic dementia, which is widely recognised as being associated with marked socio‐affective impairments (Olson et al., 2013; Thompson et al., 2003; Zahn et al., 2007). Despite this, there is a relative lack of functional neuroimaging evidence for the region's involvement in social cognition. Second, the ventrolateral ATL has been strongly implicated as a crucial site for the representation of semantic memory (or conceptual knowledge; Lambon Ralph et al., 2017) and, as we will describe in further detail below, it has been recently argued that these types of processes could account for the involvement of the ATL in social cognition (Binney & Ramsey, 2020; Olson et al., 2013). One widely regarded explanation for the ATL's role in social tasks is that it stores mental scripts, or schema, that are formed out of prior experiences and provide a wider context for understanding social interactions (Frith & Frith, 2003; Gallagher & Frith, 2003). More recent proposals have associated some ATL subregions specifically with the retrieval of social conceptual knowledge, which is posited as a subtype of semantic memory (Binney & Ramsey, 2020; Olson et al., 2013; Zahn et al., 2007). Semantic memory is a term used to refer to a long‐term store of general conceptual‐level knowledge that is involved in transforming sensory inputs into meaningful experiences, and it underpins the ability to recognise and make inferences about objects, people, and events in our environment (Lambon Ralph et al., 2017). Social conceptual knowledge has been defined distinctively as person‐specific knowledge (Simmons et al., 2010), but also knowledge about interpersonal relationships, social behaviours, and of more abstract social concepts such as *truth* and *liberty* (Olson et al., 2013; Zahn et al., 2007). Until recently, however, there has been a lack of direct evidence (e.g., from fMRI) to support the claim that the ATL is engaged in retrieving this type of information during social tasks. That is, until an influential functional neuroimaging study that revealed ATL activation that is common to both a social attribution task and a task involving semantic relatedness judgements about socially relevant concepts (Ross & Olson, 2010).

A key question arising from these accounts is whether the ATL's role in semantic retrieval is limited to social cognition, or whether it also plays a role in more general declarative memory processes. If the latter is correct, then the next question is whether this reflects a single unified set of processes or two distinct semantic systems. From one perspective, it has been suggested that social conceptual knowledge could have a special or even privileged status over other categories of semantic information (Olson et al., 2013; Zahn et al., 2007). Indeed, one influential account of the ATL, the *social knowledge hypothesis*, states that there is an ATL subregion, the dorsolateral portion (including the anterior superior and middle temporal gyri), that is selectively involved in processing social concepts (Olson et al., 2013). This account has some overlap with the *social information processing account* which claims that the dorsolateral ATL activates to social concepts as part of a larger domain‐specific network involved in social information processing (Persichetti et al., 2021; Simmons et al., 2010). Consistent with this claim are fMRI studies that demonstrate a greater response of dorsolateral ATL subregions when semantic judgements made on socially relevant stimuli, as compared to similar judgements made on non‐social stimuli (Binney, Hoffman et al., 2016; Rice et al., 2018; Ross & Olson, 2010; Zahn et al., 2007; also see Arioli et al., 2020; Lin et al., 2018; Mellem et al., 2016; Wang et al., 2019). Further, proponents of the social knowledge hypothesis argue against a more general role of ATL subregions in semantic processing, and point to the fact that a variety of socially relevant tasks and stimuli reliably activate the ATL, whereas the majority of functional imaging studies of general semantic processing do not (Olson et al., 2007, 2013; Simmons et al., 2010; Simmons & Martin, 2009).

However, the ATL is strongly implicated in general semantic processing on the basis of decades of neuropsychological data (Mion et al., 2010; Patterson et al., 2007) and a growing body of brain stimulation and electrophysiological studies, as well as functional neuroimaging studies that take special measures to address signal dropout and distortion within this region (Binney et al., 2010; Lambon Ralph et al., 2017; Visser, Embleton, et al., 2010; Visser, Jefferies, et al., 2010). A key difference between this set of literature and the majority of the ‘social concepts’ literature is that it converges on a distinct subregion of the ATL, termed the ‘ventrolateral’ ATL, which comprises the anterior fusiform and inferior temporal gyri. This therefore suggests an intriguing alternative possibility to the social knowledge hypothesis; under what might be called a ‘*dual ATL hub account*’, social conceptual knowledge and general conceptual information could be processed within distinct parts of the ATL (Zahn et al., 2007, 2009)

When broadly defined as the anterior half of the temporal lobe, the ATL is comprised of a substantial volume of cortex, amongst which there are numerous subdivisions identifiable on the basis of morphology, cytoarchitecture and connectivity (Binney et al., 2012; Ding et al., 2009; Pascual et al., 2015). Therefore, it is highly plausible that within it there are either distinct functional parcels (Persichetti et al., 2021) or graded differences in function (Binney et al., 2012; Jackson et al., 2018), including between social function and general semantic function (Binney, Hoffman et al., 2016; Olson et al., 2013). However, if hypotheses such as the dual ATL hub account are correct, then the ventrolateral ATL should not engaged by social cognitive tasks (but see Binney, Hoffman et al., 2016 ; Rice et al., 2018).

To date, the role of the ventrolateral ATL in social cognition has yet to be clearly elucidated, primarily because fMRI is the chief source of data in social neuroscience, and conventional forms of this technique are blind to activation in this region; they are vulnerable to susceptibility artefacts which cause signal loss and image distortion around the location of the ventrolateral ATL (Devlin et al., 2000). However, spin‐echo, and dual‐echo echo‐planar fMRI, as well as post‐acquisition distortion correction techniques, can be used to recover and remap signal in ventrolateral ATL (Embleton et al., 2010; Halai et al., 2014, 2015). Taking advantage of this fact, two recent studies have demonstrated that using these enhanced fMRI techniques greatly affects the patterns of activation observed across the ATL during the processing of socially relevant stimuli, and in a way that challenges the social knowledge hypothesis and dual hub account (see Binney, Hoffman et al., 2016; Rice et al., 2018). In particular, they revealed that the ventrolateral ATL activates strongly during semantic judgements made on both social and non‐social stimuli.

The studies by Binney, Hoffman et al. (2016) and Rice et al. (2018) were partially consistent with the earlier studies by Zahn et al. (2007) and Ross and Olson (2010) in that they found dorsolateral ATL activation that was selective for social semantic stimuli. However, the omni‐category response of the ventrolateral ATL was much greater in magnitude (Binney, Hoffman et al., 2016; Rice et al., 2018). The authors have argued that these observations are consistent with a third alternative account of the ATL which characterises the region as a representational substrate that is functionally unified but nonetheless reveals graded differences in semantic function. According to the *graded semantic hub* proposal, a large extent of the ATL comprises a unified representational space, all of which is engaged by the encoding and retrieval of semantic information, and by semantic information of any kind. The centre of this space exists over the ventrolateral ATL of which engagement during semantic processing is invariant to, for example, idiosyncratic task features, including the modality through which concepts are accessed. Towards the edges of this space, however, there are gradual shifts in semantic function such that regions on this periphery become relatively more specialised for encoding certain types of semantic features (Bajada et al., 2019; Binney et al., 2012; Rice, Hoffman, et al., 2015). For example, features that are primarily experienced through certain sensorimotor modalities (e.g., vision or audition; for a computational exploration of this general hypothesis, see Plaut, 2002). Along these lines, social tasks might differentially engage processing streams like those involved processing emotion‐related information. As such, the dorsolateral ATL could be sensitive to the socialness of a task because it has preferential connectivity to medial temporal limbic and frontal limbic regions (via the uncinate fasciculus; Bajada et al., 2017; Binney et al., 2012; Papinutto et al., 2016). This graded model is in direct contrast to another set of models which characterise the ATL as a patchwork of distinct functional parcels with sharp boundaries (e.g., Persichetti et al., 2021). For the purposes of the present study, however, the critical point is that, according to the graded hub account of the ATL, activation of ATL subregions in service of social cognitive tasks reflects engagement of a domain‐general semantic system and, moreover, this system is centred upon the ventrolateral ATL (Binney & Ramsey, 2020).

Regarding this proposal, the conclusions that can be drawn from Binney, Hoffman et al. (2016) and Rice et al. (2018) are limited, however. This is because they used tasks where the demands are primarily semantic in nature and the social relevance of the stimuli may have only been a secondary feature. As such, it remains an open question whether social tasks typically employed in the social neuroscience literature activate the ventrolateral ATL. The present study tackles exactly that issue, with a specific focus on mental state attribution or ‘theory of mind’ tasks. We chose this focus because ToM abilities are considered central to the construct of social cognition; they are considered as fundamental to successful social interactions, as they enable us to describe, explain and predict behaviour (Apperly, 2012; Brüne & Brüne‐Cohrs, 2006; Frith & Frith, 2005; Heleven & van Overwalle, 2018; van Hoeck et al., 2014). Neuroimaging studies reliably implicate the right temporo‐parietal junction, medial prefrontal cortex and precuneus as part of a core network for ToM (Dodell‐Feder et al., 2011; Saxe, 2006; Saxe & Kanwisher, 2003; Saxe & Wexler, 2005; Scholz et al., 2009; Young et al., 2010), whereas the role of the ATL is less clear and appears to be characterised as ancillary by some accounts (Molenberghs et al., 2016; Schurz et al., 2014; van Overwalle, 2009). It is possible that a central role of the ventrolateral ATL has gone unnoticed because fMRI studies of ToM typically do not account for technical constraints around this region.

The present study was aimed primarily at answering the following key question: whether and to what degree the ventrolateral ATL is activated by established ToM tasks. This necessitated two key design elements: (i) the use of dual‐echo fMRI and distortion correction to ensure full coverage of the bilateral ATL and (ii) the use of multiple ToM tasks. This second design feature was important because showing common activation across different ToM tasks with a variety of stimuli means that we can more confidently assess whether activation can be attributed to ToM ability itself rather than being the result of task demands or idiosyncratic features of the stimuli (Ross & Olson, 2010). In fMRI designs, the most commonly used ToM tasks include social vignettes, cartoons, and animations that are intended to evoke the attribution of intentions. We used animations as our primary task because they do not directly involve lexical‐semantic processing, which has been shown to activate the ventrolateral ATL (e.g. Binney et al., 2010; Devlin et al., 2000; Spitsyna et al., 2006; Visser, Embleton, et al., 2010; Visser, Jefferies, et al., 2010) and would confound our inferences. Moreover, they lend themselves to the creation of a comparable non‐social (and non‐semantic) control or baseline activation task, which is important for controlling for attentional and executive demands, as well as perceptual stimulation. We also acquired data during a False Belief task (Dodell‐Feder et al., 2011) and a free‐viewing animated film (Jacoby et al., 2016) as these are established paradigms for localising the ‘mentalising’ or ToM network.

As a secondary aim, we directly assessed whether ToM‐related activation includes the same ventrolateral ATL regions engaged by semantic decisions made upon nonverbal, non‐social stimuli (Visser et al., 2012). This would support the hypothesis that activation of the ATL during social tasks reflects the retrieval of semantic knowledge representations (Binney & Ramsey, 2020; Olson et al., 2013; Zahn et al., 2007). While involvement of this region in general semantic processing is well established (see above), there has yet to be a direct demonstration that both ToM tasks and semantic tasks engage this same region. We used the picture version of the Camel and Cactus task (CCT), which is an established means to engage and measure non‐verbal semantic processing, and has been previously used in neuropsychological, functional imaging and brain stimulation studies (Bozeat et al., 2000; Hoffman et al., 2012; Jefferies & Lambon Ralph, 2006; Visser et al., 2012).

Finally, using these data, we were able to test predictions derived from three different accounts of ATL function, as follows:

1. The *Social Knowledge Hypothesis/Social Information Processing Account*: according to these accounts, the ATL is part of a domain‐specific network involved in processing social information (Simmons et al., 2010), and the dorsolateral ATL is selectively involved in processing concepts with social–emotional content (Olson et al., 2013). Proponents of this account argue against a domain‐general role of the ATL in semantic representation (Olson et al., 2013; Simmons et al., 2010). If this is correct, then the ATL would activate for ToM tasks but not during the CCT.
2. The *Dual ATL Hub account*: if a dual hub account is correct then the ToM tasks would exclusively activate the dorsolateral/polar ATL and not the ventrolateral aspect, and the CCT would only activate the ventrolateral ATL.
3. The *Graded ATL Semantic Hub Hypothesis*: if the third account, in which the ventral ATL is the centre point of a domain‐general hub for both social and non‐social semantic processes (Binney, Hoffman et al., 2016), is correct, then all three of the ToM tasks and the CCT will engage the ventrolateral ATL. We might also observe dorsolateral activation that is more selective to social stimuli.

## 2 MATERIALS AND METHODS

### 2.1 Design considerations

To ensure that the imaging protocol was sensitive to changes in activation across all parts of the ATL, we used a novel combination of methods for recovering and remapping blood oxygen level‐dependent (BOLD) signal in areas that are usually prone to magnetic susceptibility‐induced artefacts. First, we used a dual‐echo gradient‐echo echo‐planar imaging (EPI) fMRI sequence that is optimised for the ATL and has been shown to have greater ability to detect inferior temporal lobe activation compared to single‐echo GE‐EPI sequences with a conventional echo time around 30 ms (Embleton et al., 2010; Halai et al., 2014, 2015). However, the dual‐echo method advocated by Halai and colleagues does not correct for the impact of geometric distortions, which occur within the images acquired at the shorter echo‐time and are also caused by magnetic susceptibility artefacts. These distortions have the potential to reduce signal/contrast‐to‐noise ratios as well as result in mislocalisations of task‐related activity changes. Therefore, in the present study, the dual‐echo sequence was combined, for the first time, with a post‐acquisition k‐space spatial correction. Our choice of distortion correction procedure was based on evidence that that it outperforms more standard corrections that use phase‐encoded field maps (Embleton et al., 2010). We also made other methodological decisions such as acquiring with a left‐to‐right phase‐encoding direction that contributes to better quality of data in the inferior ATL (Embleton et al., 2010). To demonstrate the effectiveness of these measures, we followed the procedure of previous reports investigating the ATLs (Hoffman et al., 2015; Simmons & Martin, 2009) and calculated an average map of temporal signal‐to‐noise ratio (tSNR) (see Supplementary Figure M1). The tSNR map suggests a good signal quality even in the in most ventral proportions of the ATL.

Secondly, we adapted stimuli created by Walbrin et al. (2018) to fashion a two alternative force choice (AFC) task involving explicit judgements about the intention of moving shapes (are they behaving in ‘friendly’ or ‘unfriendly’ way?) that are seemingly engaged in goal‐directed action. Our task is broadly inspired by the widely used Frith‐Happé animations for probing ToM processes (see e.g., Abell et al., 2000; Bliksted et al., 2019; Castelli, 2002; Das et al., 2012; Hennion et al., 2016; Kana et al., 2009; Synn et al., 2018), but it was designed to be closer to that used in a key study by Ross and Olson (2010); (also see Schultz et al., 2003). We specifically chose Walbrin and colleagues' stimuli because they offer a higher number of unique trials (impacting sensitivity/power) than other similar stimuli sets, and they are visually well controlled to minimise the contribution of low‐level visual information to brain responses (i.e., they are comprised of visually diverse interactive scenarios that are well matched for overall motion energy). To control for attentional and executive demands involved in the main task, we reconfigured these stimuli to further create a well‐matched perceptual judgement task. See below for further detail regarding the experimental and control task.

### 2.2 Participants

Thirty‐one healthy native English speakers took part in the experiment. All participants had normal or corrected‐to‐normal vision, no history of neurological and psychiatric conditions and were right‐handed as established by the Edinburgh Handedness Inventory (Oldfield, 1971). Participants provided written informed consent, and the study was approved by the local research ethics review committee. Seven participants were excluded because of inadequate task performance (under 70% accuracy) on any one of the social interaction tasks (*N* = 3), or because of failed distortion correction and therefore insufficient data quality (*N* = 4). The final analysed sample comprised of 24 participants (12 females, *M*_age_ = 22.21, *SD*_age_ = 2.13).

### 2.3 Experimental stimuli and tasks

#### 2.3.1 Theory of mind

A total of 126 unique video stimuli designed by Walbrin et al. (2018) were used for the main interaction judgement theory of mind (IJ‐ToM) task and its corresponding control task. The IJ‐ToM stimuli (*N* = 63) featured two self‐propelled circles representing animate agents that were intentionally interacting and doing so in a co‐operative manner in half the trials and a competitive manner in the other half (see Supplementary Figure M2). In Walbrin and colleagues' original stimulus pool (*N* = 256) half of the scenarios concluded with successful goal outcome (e.g., successfully opening a closed door) and half with unsuccessful goal outcomes. Here, we only used a subset of the former. In the IJ‐ToM task, participants were instructed to make explicit inferential judgements, via a key press, as to whether the agents' actions towards one another were friendly or unfriendly (unlike the original study that sought to minimize the contribution of ToM judgements and employed a perceptual response task). The associated control task used ‘scrambled’ versions of the interaction stimuli (*N* = 63) that preserved many of the visual properties but featured altered motion paths such that the shapes did not appear to be intentionally interacting with each other or their environment (see Walbrin et al., 2018). For the present study, these control stimuli were adjusted such that in 50% of trials the speed of motion of one of the two shapes was slower than that of the other. This was done by slowing the frame rate of one of the animation elements (i.e., one of the circles) from 24 to 18 frames per second and removing frames from either the beginning (50%) or end of the sequence to maintain the original duration (6 s). Moreover, we ensured that the more slowly moving object appeared an equal number of times at each relative position on the screen (e.g., left vs. right). Participants responded to these stimuli via key press and indicated whether they believed the circles were moving at same or different speeds. Following some initial pilot behavioural testing, the duration of all 126 videos was shortened from 6 to 3 s to increase task difficulty/eliminate idle time.

We also acquired data with two widely used functional localisers for the putative ToM network, namely the False Belief (FB) paradigm (Dodell‐Feder et al., 2011) and a more recently validated free‐viewing movie (MOV) paradigm (Jacoby et al., 2016). The former is a verbal paradigm, which is comprised of two sets of 10 text‐based vignettes each of which are presented on screen and followed by true/false questions. One of these sets requires the participant to make inferences about a character's internal beliefs and this is contrasted against descriptions of facts about physical events. The MOV paradigm involves passive viewing of a commercial animated film and contrasts BOLD responses to events in which characters are involved in ToM against those in which characters experience physical pain (see Jacoby et al., 2016 for more detail).

#### 2.3.2 Non‐verbal semantic association

Participants also completed a non‐verbal version of an established neuropsychological assessment of semantic associative knowledge known as the Camel and Cactus task (CCT; Bozeat et al., 2000). This task has been used to engage the semantic network in prior fMRI studies (Rice et al., 2018; Visser et al., 2012). The version used in the present study consisted of 36 trials that contained pictorial stimuli and required participants to make semantic associations between a probe object (e.g., a camel) and a target object (e.g., a cactus) that was presented alongside a foil from the same semantic category (e.g., a rose). The CCT was contrasted against a perceptual control task (36 trials) that consisted of scrambled versions of the CCT pictures and required participants to identify which of two choice pictures was visually identical to a probe (see more detail in the study by Visser et al., 2012).

### 2.4 Experimental procedure

Participants underwent all testing within a single session lasting approximately 1 h. Each individual completed three runs of the IJ‐ToM procedure reported below, followed by one run of the CCT procedure, two runs of the FB localiser and one run of the MOV localiser. The IJ‐ToM task, the CCT and FB task and the corresponding control tasks were presented via E‐prime (Psychology Software Tools, 2017) and the MOV task via Psychtoolbox (Brainard, 1997; Pelli, 1997) software. Behavioural responses were recorded using an MRI compatible response box.

#### 2.4.1 Interaction judgement ToM task

The IJ‐ToM task and the speed judgement task were paired within a run using an A‐rest‐B‐rest box car block design. Each run contained six blocks per task and three trials per block (18 trials per run per task). There were an equal number of trial types (e.g., *cooperative* versus *competitive*) randomly distributed across blocks within a given run. Both types of active blocks were 17.25 s long and they were separated by blocks of passive fixation lasting 12 s each. Each trial began with a fixation cross (duration = 500 ms) which was followed by the target animation (3000 ms) and finished with a response cue (three question marks; 2000 ms). A blank screen occupied an inter stimulus interval of 250 ms. Each run lasted 5 min and 51 s and consisted of unique sets of animations. The order in which these runs were completed was counterbalanced across participants. Participants also completed three practise blocks for each of the two tasks before the main runs began.

#### 2.4.2 Camel and Cactus task

The CCT and the corresponding perceptual identity matching control task were alternated within a single run using a blocked design. There were nine blocks per task, each consisting of four trials (totalling 36 trials per task) and lasting 20 s. A trial began with a fixation cross (500 ms) followed by a stimulus triad (4500 ms). Participants responded via key press while the probe and choice items were on screen. Active blocks were separated by brief rest blocks lasting 4000 ms and, overall, the run lasted for 7 min and 12 s.

#### 2.4.3 False belief and animated movie localisers

Each run of the false belief localiser lasted 4 min and 32 s and consisted of 10 trials of belief vignettes and 10 trials of the fact vignettes. Finally, the passive MOV scanning run lasted 5 min and 59 s including a fixation period of 10 s prior to the beginning of the movie. Further details regarding these paradigms are reported by Jacoby et al. (2016).

### 2.5 Imaging acquisition

All imaging was performed on a 3 T Phillips Achieva MRI scanner with a 32‐element SENSE head coil using a 2.5 sense factor for image acquisition. The parameters of the dual‐echo gradient‐echo EPI fMRI sequence were the following: 31 axial slices covering the whole brain and obtained in an ascending sequential order with a first echo time (TE) = 12 ms and second TE = 35 ms, repetition time (TR) = 2000 ms, flip angle = 85°, FOV (mm) = 240 × 240 × 124, slice thickness = 4 mm, no interslice gap, reconstructed voxel size (mm) = 2.5 × 2.5 and reconstruction matrix = 96 × 96. Prior to image acquisition for each run, we acquired five dummy scans to allow the initial magnetisation to stabilise. This was followed by acquiring 177 volumes for each IJ‐ToM task run, 218 volumes for the CCT task run, 138 volumes for each FB task run and 180 volumes for the MOV task run. Adhering to the distortion‐correction method, we acquired these functional runs with a single direction k space traversal in the left–right phase‐encoding direction. We also acquired a short EPI ‘pre‐scan’ with the participants at rest. The parameters of the pre‐scan matched the functional scans except that it included interleaved dual direction k space traversals. This gave 10 pairs of images with opposing direction distortions (10 left–right and 10 right–left) which were to be used in the distortion correction procedure described below. To check the quality of the distortion corrected images, we obtained a high‐resolution T2‐weighted scan consisting of 36 slices covering the whole brain, with TR = 17 ms, TE = 89 ms; reconstructed voxel size (mm) = 0.45 × 0.45 × 4; reconstruction matrix = 512 × 512. Additionally, we used a T1‐weighted 3D imaging sequence to acquire an anatomical scan, consisting of 175 slices covering the whole brain, for use in spatial normalisation procedures. The parameters of this scan were as follows: P reduction (RL) SENSE factor of 2 and S reduction (FH) SENSE factor of 1, TR = 18 ms, TE = 3.4 ms, 8° flip angle, reconstructed voxel size (mm) = 0.94 × 0.94 × 1.00 and reconstruction matrix = 240 × 240.

### 2.6 Data analysis

#### 2.6.1 Behavioural data

Incorrectly answered trials, missed trials and trials with response latencies that were 2 SD above or below the participant's task mean were excluded from analyses of behavioural data. Task performance was assessed in terms of both accuracy and decision times and compared using paired‐sample T‐tests. Average decision times per block of each task were also calculated so that they could be used as regressors of no interest in fMRI analyses.

#### 2.6.2 Distortion correction and fMRI pre‐processing

A spatial remapping correction was computed separately for images acquired at the long and the short echo time and using a method reported elsewhere (Embleton et al., 2010). This was implemented via in‐house MATLAB script (available upon request) as well as SPM12's (Statistical Parametric Mapping software; Wellcome Trust Centre for Neuroimaging, London, UK) 6‐parameter rigid body registration algorithm. Briefly, in the first step, each functional volume was registered to the mean of the 10 pre‐scan volumes acquired at the same echo time. Although this initial step was taken primarily as part of the distortion correction procedure, it also functioned to correct the time‐series for differences in subject positioning in between functional runs and for minor motion artefacts within a run. Next, one spatial transformation matrix per echo time was calculated from opposingly distorted pre‐scan images. These transformations consisted of the remapping necessary to correct geometric distortion and were applied to each of the main functional volumes. This resulted in two motion‐ and distortion‐corrected time‐series per run (one per echo), which were subsequently combined at each timepoint using a simple linear average of image pairs.

All of the remaining pre‐processing steps and analyses were carried out using SPM12. Slice‐timing correction referenced to the middle slice was performed on the distortion‐ and motion‐corrected images. The T1‐weighted anatomical scan was co‐registered to a mean of the functional images using a six‐parameter rigid‐body transform, and then SPM12's unified segmentation and normalisation procedure and the DARTEL (diffeomorphic anatomical registration though an exponentiated lie algebra; Ashburner, 2007) toolbox were used to estimate a spatial transform to register the structural image to Montreal Neurological Institute (MNI) standard stereotaxic space. This transform was subsequently applied to the co‐registered functional volumes, which were resampled to a 3 × 3 × 3 mm voxel size and smoothed with an 8 mm full‐width half‐maximum Gaussian filter.

#### 2.6.3 fMRI statistical analysis

Data were analysed using the general linear model approach (GLM). At the within‐subject level, a fixed effect analysis was carried out upon each task pair (e.g., the interaction judgement task and the perceptual control task), incorporating all functional runs within a single GLM. Block onsets and durations were modelled with a boxcar function and convolved with the canonical hemodynamic response function. A high‐pass filter with a cut off of 128 s was also applied. The extracted motion parameters were entered into the model as regressors of no interest. Decision time data were also modelled to account for differences in task difficulty. Due to the block design employed, there was a single value for each epoch of a task which was the average of response times across the trials. These average decision times for each block were mean centred. To avoid false positive activations in the surrounding CSF due to physiological noise, we used an explicit mask restricted to cerebral tissue that was created from tissue segments generated by DARTEL in MNI space and binarised with a 0.4 threshold.

At the level of multi‐subject analyses, we first examined activation during the IJ‐ToM task at the whole brain level. To complement the whole‐brain topographic representation of the data, we used a priori regions of interest (ROI), defined on the basis of independent datasets, to extract and quantify the magnitude of activation within different ATL subregions. This was done using the SPM MarsBar toolbox (Brett et al., 2002). A key aim of this study was also to assess whether parts of the ATL are commonly activated by different types of behavioural paradigm used to localise the putative ToM network. To do this, we performed a formal conjunction analysis (Nichols et al., 2005; Price & Friston, 1997). In addition to our interacting geometric shapes paradigm, this included a version of False Belief task (Dodell‐Feder et al., 2011) which is comprised of verbal vignettes, and a free‐viewing movie paradigm (Jacoby et al., 2016). This analysis was an important step because it would enable us to home in on those regional activations that are a feature of ToM abilities irrespective of the manner in which they are probed. Moreover, if one is to compare ToM tasks that are qualitatively very different from one another, it is possible to attribute the common activations much more convincingly to the particular cognitive process of interest, as opposed to similarities in physical stimulus properties or peripheral elements of the task demands (Friston et al., 1999). Using a further conjunction analysis, we also explored overlap between the IJ‐ToM task and a nonverbal semantic association task. This enabled us to test the hypothesis that activation of the ATL during social tasks reflects the retrieval of semantic knowledge representations (Binney & Ramsey, 2020; Olson et al., 2013; Zahn et al., 2007).

Whole‐brain multi‐subject random effects analyses were conducted on each of the following contrasts of interest: IJ‐TOM task: interaction > speed judgements, interaction judgements > rest, speed judgements > interaction judgements; CCT task: semantic > perceptual judgements; FB task: false belief > false fact judgements; MOV task: mentalizing > pain. One‐sample *t*‐tests were performed on all sets of contrast images following application of the same explicit mask as used in the single subject analyses. The resulting statistical maps were assessed for cluster‐wise significance using a cluster‐defining voxel‐height threshold of *p* < .001 uncorrected, and family‐wise error (FWE) corrected cluster extent threshold at *p* < .05 (calculated per SPM12 under the random field theory framework; see details regarding smoothness of data, the search volumes and RESELS in Supplementary Table M1). Thresholded maps were overlaid on a MNI152 template brain using MRIcroGL (https://www.nitrc.org/projects/mricrogl). We used an AAL atlas implemented in R label4MRI package (https://github.com/yunshiuan/label4MRI) to guide the labelling of peak co‐ordinates in the output tables. To maintain objectivity regarding what is and what is not considered ATL activation, we used an ATL definition used by Rice, Hoffman, et al. (2015). Rice and colleagues defined a plane perpendicular to the long axis of the temporal lobe (passing through the fusiform gyrus at *y* = −20, *z* = −30 and STG at *y* = 0, *z* = −5) and classified all temporal lobe peaks anterior to this plane as falling within the ATL.

**FIGURE 1:**
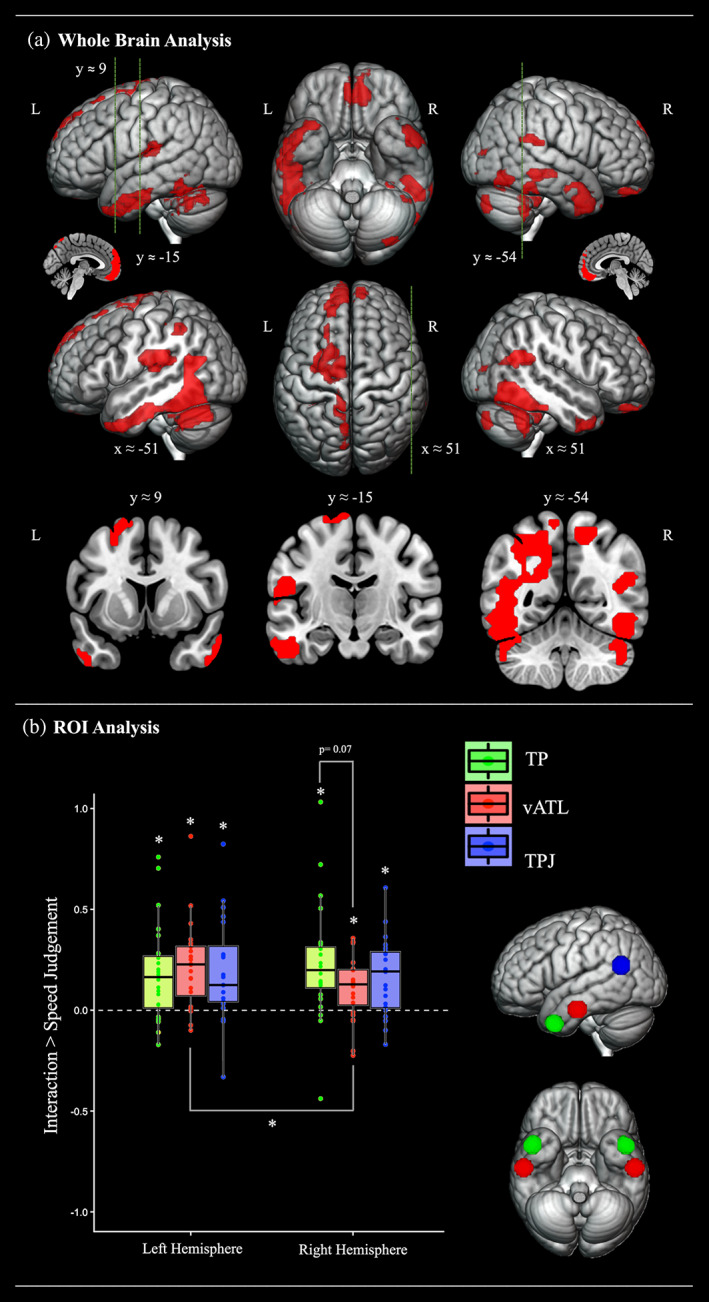
(a) Whole brain analysis. Cortical regions activated during the main experimental ToM task (the interaction judgement), relative to the speed judgement control task. The statistical map was thresholded with an uncorrected voxel height threshold of *p* < .001 and a family wise error corrected minimum cluster extent threshold (*k* = 152) at *p* < .05. Cross sections were chosen to display the location of activation found in key studies investigating ToM processing (Saxe & Kanwisher, 2003; right TPJ [51, −54, 27]), semantic processing of social concepts (Binney, Hoffman, & Lambon Ralph, 2016; left TP [−48, 9, −39]) and general semantic processing (Visser et al., 2012; left inferior ATL [−57, −15, −24]). (b) ROI analysis. summary of the ROI analyses comparing the magnitude of activation for the interaction judgement ToM task (relative to that during speed judgements control task). An asterisk denotes a significant effect at *p* < .05 after Bonferroni correction. Numerical *p* values are displayed where comparisons yielded a *p* value greater than .05 but less than .1. L, left; R, right; TP, temporal pole; TPJ, temporo‐parietal junction; vATL, ventrolateral anterior temporal lobe

Within the ROI analysis (Brett et al., 2002), two ATL subregions were explored in each hemisphere. A ventrolateral ATL ROI was defined by peak coordinates of activation reported by an independent study of non‐verbal semantic processing that used the same semantic association (CCT) task (Visser et al., 2012) (MNI: ±57, −15, −24). We chose this definition (a) with a view to confirm the findings of Visser et al. (2012) and (b) because both the principal ToM task and the general semantic task contain nonverbal stimuli, and stimulus modality has been shown to modulate the topology of ATL activation in semantic tasks (Rice, Hoffman, et al. 2015). We also examined a polar ATL ROI which was defined on the basis of activation tuned towards socially relevant semantic stimuli as reported by Binney, Hoffman et al. (2016) (MNI: ±48, 9, −39). We decided to use this ROI definition as opposed to using more dorsally located coordinates from Zahn et al. (2007), because Binney, Hoffman et al. (2016) used a more tightly controlled set of social and non‐social semantic stimuli. Furthermore, so that we could compare the degree of ATL activation to that of a more established ToM region, we defined a third ROI on the basis of ToM‐related TPJ activation reported by Saxe and Kanwisher (2003) (±54, −60, 21). These sets of coordinates defined a centre of mass for spheres with a radius of 10 mm (See Figure 1b for an illustration of ROI locations). Per subject, a single summary statistic was calculated to represent activation across all the voxels in an ROI (the mean of the parameter estimates) for the IJ‐ToM task relative to the speed judgement control task. One‐sample *t*‐tests were then performed to assess group‐level significance. To control for multiple comparisons, *p* values were Bonferroni corrected on the basis of the number of ROIs (multiplied by 6) as implemented in MarsBar. We also conducted planned comparisons between ROIs in each hemisphere and between hemispheric homologue regions, using paired *t*‐tests. For the conjunction analyses, we used a *p* < .001 uncorrected voxel height threshold to be achieved by each contrast independently prior to conjunction (Nichols et al., 2005; Price & Friston, 1997).

## 3 RESULTS

### 3.1 Behavioural data

Mean accuracy and decision times for all tasks are displayed in Table 1. Performance on the animated interaction friendliness judgement (IJ ToM task) was more accurate than on the speed judgement control task (*t*(23) = 7.50, *p* < .001, Cohen's *d* = 1.53), and decision times were also faster (*t*(23) = −3.08, *p* = .005, *d* = 0.63). Performance during semantic association judgements (CCT task) was less accurate than performance in the perceptual identity matching control task (*t*(23) = −8.83, *p* < .005, *d* = −1.80), although there was no significant difference in the latency of decision times (*t*(23) = −0.65, *p* = .522, *d* = −0.13). Accuracy across the false belief and false facts judgements (FB task) was comparable (*t*(23) = 0.77, *p* = .450, *d* = 0.16) although decision times were faster in the false fact task (*t*(23) = 2.73, *p* = .012, *d* = 0.56).

**TABLE 1:** Behavioural data

Task	Accuracy (%)	Decision time (ms)
Interaction Friendliness Judgement	96.37 (04.35)	468.38 (125.44)
Perceptual Speed Judgement	84.88 (07.62)	524.25 (159.84)
Semantic Association Judgement	79.75 (17.84)	1491.92 (382.65)
Perceptual Identity Matching	92.59 (19.96)	1529.76 (460.57)
False Belief Judgement	70.42 (19.67)	2779.56(385.47)
False Fact Judgement	67.92 (15.87)	2560.31(354.64)

*Note*: Standard deviations stated in parentheses.

### 3.2 Activation during a social attribution task given full temporal lobe coverage

A whole brain univariate analysis contrasting social interaction friendliness judgements with the matched speed judgement task revealed robust bilateral ATL activation that was centred over the ventrolateral aspects in both hemispheres (see Figure 1a and Table 2). In the left hemisphere, this extended from the ventrolateral temporopolar cortex (BA38), along the inferior middle temporal gyrus and inferior temporal gyrus (ITG), to approximately halfway along the temporal lobe (*y* ≈ −17). This included a maxima that is notably similar in location (MNI coordinates *x* = −54, *y* = 6, *z* = −39) to that identified in association with processing of abstract social concepts (relative to matched abstract non‐social concepts; *x* = −54, *y* = 9, *z* = −33 and animal function concepts; *x* = −48, *y* = 9, *z* = −39) by Binney, Hoffman et al. (2016). The same cluster also extended more posteriorly upon the basal surface and along the fusiform/lingual and posterior inferior temporal gyri. It also traversed up into the parietal lobe and the intraparietal sulcus. In the right hemisphere, ATL activation also covered much of the ventrolateral surface [particularly the polar cortex and the anterior‐most portion of the middle temporal gyrus (MTG) but extended less posteriorly (to y ≈ −11) than it did in the left].

**TABLE 2:** Significant activation clusters in the social interaction judgement > speed judgement contrast (*p* < .05, FWE‐corrected, corresponding to an extent threshold of *k* = 152 following a cluster‐defining threshold of *p* < .001, uncorrected)

Cluster name and location of maxima	Cluster extent (voxels)	Peak (Z)	MNI coordinates (mm)		
			*x*	*y*	*z*
L temporal–parietal–occipital	2242				
Anterior ITG/sulcus		5.72	−57	−6	−30
Anterior ITG		5.26	−54	6	−39
Posterior ITG		5.07	−51	−51	−24
Precuneus		4.78	−12	−60	42
Posterior ITG		4.76	−45	−60	−9
Inferior parietal lobule		4.68	−39	−51	45
Precuneus		4.49	−9	−75	48
Middle/anterior ITG		4.42	−48	−21	−27
Posterior MTG		4.23	−48	−54	3
Cerebellum		4.21	−51	−51	−36
Posterior MTG		4.10	−39	−60	15
Anterior MTG/TP		4.04	−42	18	−42
R temporal–parietal–occipital	1708				
Inferior occipital gyrus		5.52	48	−63	−12
Occipital pole		5.01	30	−90	−6
Middle occipital gyrus		4.74	33	−75	3
Posterior MTG		4.39	63	−36	−9
Cerebellum		4.35	36	−42	−33
Cerebellum		4.29	27	−78	−42
Cerebellum		4.28	36	−84	−39
Posterior STG/TPJ		4.26	57	−39	21
Cerebellum		4.24	42	−48	−30
Posterior STS/TPJ		4.22	42	−60	18
Cerebellum		4.21	45	−51	−42
Middle occipital gyrus		4.05	39	−81	12
Bilateral frontal	1017				
L anterior SFG		5.46	−3	63	21
R anterior orbital gyrus		5.30	9	45	−24
R anterior gyrus rectus		5.21	6	51	−18
L middle SFG		5.08	−12	60	27
L middle SFG		4.93	−12	57	36
R middle SFG		4.81	3	60	30
R anterior mPFC		4.77	9	63	−9
R anterior mPFC		4.77	3	57	−6
R anterior mPFC		3.45	3	60	6
L temporal–parietal	362				
Superior parietal lobule		4.65	−54	−27	18
Middle STG		3.60	−63	−15	9
R anterior temporal	160				
Anterior MTG		4.57	60	6	−33
Anterior ITG		4.54	51	12	−45
Anterior MTG/TP		3.97	51	18	−39
Anterior MTG/TP		3.54	45	24	−39
Anterior MTG		3.37	60	−9	−24
L dorsal frontal	410				
Middle superior frontal sulcus		4.38	−24	3	60
Posterior superior frontal sulcus		4.29	−24	0	48
Posterior SFG		4.15	−12	−12	78
Posterior superior frontal sulcus		4.01	−30	−6	63
Posterior SFG		3.30	−21	27	60
R parietal	152				
Superior postcentral gyrus		4.29	24	−42	57
Superior parietal lobule		4.09	18	−57	60
Precuneus		3.32	9	−63	54

*Note*: The table shows up to 12 local maxima per cluster more than 8.0 mm apart.

Abbreviations: AG, angular gyrus; ITG, inferior temporal gyrus; L, left; MTG, middle temporal gyrus; mPFC, medial frontal cortex; R, right; SFG, superior frontal gyrus; STS, superior temporal sulcus; TP, temporal pole; TPJ, temporo‐parietal junction.

Outside of the ATL, and as expected, this contrast also revealed activation amongst key nodes of the putative ToM network, including the temporoparietal junction (TPJ), the medial prefrontal cortex (mPFC) and the precuneus (Frith & Frith, 2003; Frith & Frith, 2006; Jacoby et al., 2016; Saxe & Kanwisher, 2003; van Overwalle, 2009). TPJ activation was observed in both hemispheres at the position of the posterior superior temporal sulcus/gyrus (STS/STG) and, in the right hemisphere, it extended to more posterior regions (at *y* ≈ −54) that are frequently emphasised in landmark studies (Saxe & Kanwisher, 2003; Saxe & Powell, 2006) and large‐scale meta‐analyses (Molenberghs et al., 2016; Schurz et al., 2014) of the ToM network. Further activation was revealed in the left posterior MTG, the left insula, and bilateral temporooccipital and cerebellar regions.

Activation during the social interaction friendliness judgements was also contrasted with passive fixation/rest. There was notably little activation in the ATLs, except for a small cluster in the left superior temporal pole (see Supplementary Figure S1 and Table R1). This is consistent with the idea that there may be automatic semantic activation (e.g., mind‐wandering, episodic recall and socially oriented thoughts) during periods of passive fixation, and it demonstrates the importance of using active baseline tasks for detecting ATL activation which has been highlighted in prior meta‐analyses and empirical investigations (Andrews‐Hanna et al., 2014; Binder et al., 1999, 2009; Visser, Embleton, et al., 2010; Visser, Jefferies, et al., 2010).

Outside of this region there was robust bilateral fronto‐parietal activation including of the bilateral TPJ and the ventrolateral prefrontal cortex, and activation of the mPFC, the precuneus, and temporooccipital and cerebellar regions. The contrast revealing greater activation for the speed judgement relative to the social attribution task is reported in Supplementary Figure S2 and Table R2 and revealed the right middle frontal gyrus and a number of midline structures.

We used an a priori ROI‐based approach to compare the magnitude of regional responses to the social attribution task both within each hemisphere and between hemispheric homologues. We focused upon two key ATL subregions, the temporopolar cortex and the posteriorly adjacent ventrolateral surface, as well as temporoparietal cortex (i.e., the TPJ) frequently implicated in ToM. The positions of these ROIs and the results are displayed in Figure 1b. Bonferroni‐corrected one‐sample T‐tests revealed significant activation during social interaction judgements in the left vATL (*t*(24) = 5.09, Cohen's *d* = 1.04), temporal pole (*t*(24) = 3.77, Cohen's *d* = 0.77) and TPJ (*t*(24) = 3.94, Cohen's *d* = 0.80) and also the right vATL (*t*(24) = 3.57, Cohen's *d* = 0.73), temporal pole (*t*(24) = 4.03, Cohen's *d* = 0.82) and TPJ (*t*(24) = 4.41, Cohen's *d* = 0.90) (all *p* < .005). Numerically speaking, across all the ROIs, the left vATL revealed the largest effect size, and the TPJ showed the weakest effects. Planned statistical comparisons (see Supplementary Table R3) confirmed greater activation in the left as compared to the right vATL (*t*(24) = 2.45, *p* = .02, Cohen's *d* = 0.50). There were no other significant pairwise differences.

### 3.3 Common activation of the ATL across three different ToM paradigms

**FIGURE 2:**
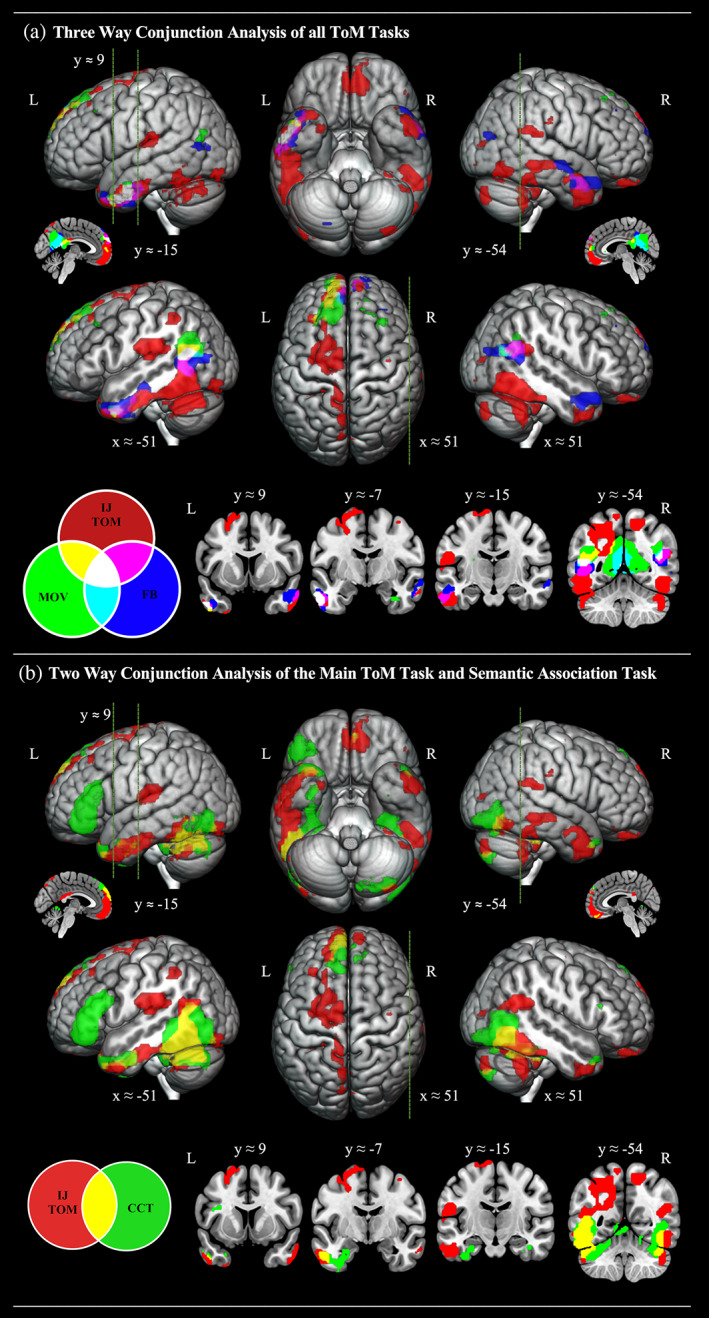
(a) Topological overlap of cortical regions activated by the interaction judgement > speed judgement contrast, the false belief story > photograph contrast, and the mentalising > pain contrast from the free‐viewing movie localiser. Each of the three statistical maps was independently thresholded with an uncorrected voxel height threshold of *p* < .001 and then overlaid within MRICron using additive colour blending. White patches indicate three‐way overlap between all three ToM contrasts. (b) Topological overlap of cortical regions activated by the interaction judgement > speed judgement contrast (red), and the nonverbal semantic association (Camel and Cactus task) > perceptual judgement contrast (green). The two statistical maps were independently thresholded with an uncorrected voxel height threshold of *p* < .001 and then overlaid within MRICron using additive colour blending. Yellow patches indicate overlap between theory of mind and general semantic processing. Cross sections were chosen to display the location of activation found in key studies investigating ToM processing (Saxe & Kanwisher, 2003; right TPJ [51, −54, 27]), semantic processing of social concepts (Binney, Hoffman, & Lambon Ralph, 2016; left TP [−48, 9, −39]) and general semantic processing (Visser et al., 2012; left inferior ATL [−57, −15, −24]), as well one further key area of 3‐way overlap (*y* = −7)

In the subsequent analysis, we aimed to map out subregions of the bilateral ATL in which there is overlapping activation between some of the different types of behavioural paradigm used to localise the putative ToM network (Dodell‐Feder et al., 2011; Jacoby et al., 2016). The results of independent whole‐brain analyses contrasting two further ToM tasks (the False Belief task and the free‐viewing movie paradigm) with their respective control tasks are reported in Supplementary Figures S3 and S4 and Tables R4 and R5. We formally assessed activation overlap between the three ToM tasks using a conjunction analysis performed across the whole brain (Nichols et al., 2005). For complete visualisation of the results and to capture the full extent of both the overlap and divergence in the topography of activation, the three whole brain activation maps are overlaid on each other in Figure 2a, whereas a map limited to the formal statistical conjunction can be found in Supplementary Figure S5 and Table R6. Regarding ATL activation, the conjunction analysis revealed three‐way overlap between the ToM tasks exclusively within the left ventrolateral ATL. This extended over the anterior ITG and MTG from about *y* ≈ −7 to *y* ≈ 9 and is also strikingly similar to ATL regions reported as activated by social concepts by Binney, Hoffman et al. (2016). As would be expected from prior literature, three‐way overlap was also observed in the mPFC and bilateral TPJ.

### 3.4 ATL activation common to both ToM and general semantic processing

Finally, we performed a conjunction analysis aimed at identifying any potential overlap between ATL regions engaged by ToM tasks and those engaged by general semantic processing. The same sample of participants and the same ATL‐optimised dual‐echo imaging sequence were used to acquire fMRI data while individuals completed a nonverbal semantic association task. The result of an independent whole‐brain analysis contrasting this task with a matched control task is reported in Supplementary Figure S6 and Tables R7. In the ATL, there was a cluster stretching from the left TP all the way to the ventrolateral aspects. Outside the anterior temporal regions, we observed activation in the posterior occipitotemporal areas bilaterally and left pMTG and IFG. The left SFG was also activated on the medial surface. This contrast was entered into a whole brain conjunction analysis along with the interacting geometric shapes paradigm. The full extent of overlap and divergence between ToM activation and general semantic activation is displayed in Figure 2b, while the results of the formal statistical conjunction are found in Supplementary Figure S7 and Table R8. Both ToM and general semantics activated the left ventrolateral ATL. Specifically, there was a cluster of 114 commonly activated voxels in the left ventral ATL with the activation starting to converge at *y* ≈ −15, showing the most robust overlap at *y* ≈ −7, and still overlapping in inferior polar regions at *y* ≈ 9. There was a further common ATL activation (extent = 32 voxels) within the left medial temporal pole. On the basis of this analysis, the right ATL appeared only to be activated by the IJ‐ToM task. Outside of the ATL region, there was also overlap in the left pMTG and TPJ region, as well as the left mPFC and bilateral inferior temporo‐occipital regions.

## 4 DISCUSSION

The present study was aimed at establishing whether the ventrolateral surface of the anterior temporal lobe (ATL), a region strongly implicated in general semantic processing, is engaged during ToM tasks. We also directly compare the topology of ATL activation associated with ToM with that evoked by a nonverbal non‐social semantic task. The results have implications for theories regarding the functional role of the broader ATL region in social cognition, and they can be used to evaluate hypotheses derived from three alternative accounts. According to the *social knowledge hypothesis*, the ATL serves a domain‐specific role in processing socially relevant semantic information. Proponents of this account have argued against any role of ATL regions in domain‐general semantic processing (Olson et al., 2013; Simmons et al., 2010). Another hypothesis, which we refer to as the ‘*dual hub*’ account, distinguishes between two separate ATL hubs, one for social processing and one for general semantic processing. Alternatively, the *graded semantic hub* hypothesis holds that the ATL is a unified domain‐general conceptual hub involved in the representation of all manner of conceptual‐level knowledge (Binney, Hoffman et al., 2016; Lambon Ralph et al., 2017). According to this account, the ventrolateral ATL is a critical centre‐point for general semantic knowledge representation and, as such, will be activated by all manner of semantically imbued tasks or behaviour. The key findings of the present study were as follows:

1. By using distortion‐corrected dual‐echo fMRI, we were able to confirm, within a whole‐brain analysis, that the bilateral ventrolateral ATL (inclusive of the anterior ITG and MTG) is engaged by a nonverbal task of the kind that has frequently been employed to localise the putative ToM network. A region of interest analysis revealed that the left ventrolateral ATL was more activated than the right homologue, and both these regions were as robustly activated as the TPJ, which is another key node in the ToM network.
2. Moreover, left ventrolateral ATL (anterior ITG and MTG) activation was confirmed as a key feature of ToM by the fact that it was activated robustly across three different paradigms employing a range of verbal and nonverbal stimuli.
3. Finally, the left ATL activation associated with ToM overlapped with that evoked by semantic association judgements performed on non‐social picture stimuli, specifically within the anterior ITG.

These findings confirm the role of the ventrolateral ATL in ToM tasks. Further, they are, overall, most consistent with the hypothesis that the ATL is a domain‐general conceptual hub and suggest that its contribution to social cognition is related to the retrieval of a general class of semantic knowledge representations (Binney & Ramsey, 2020). These findings are not compatible with the social knowledge hypothesis or the dual hub hypothesis.

### 4.1 The functional contribution of ATL subregions to social and semantic cognition

A link between certain parts of the ATL (e.g., temporopolar cortex; for a review, see Olson et al., 2013) and social cognition has been recognised for well over a century, owed in part to the acclaimed work of Brown & Sharpey‐Schafer, 1888 and, later, Klüver and Bucy (1937) who performed bilateral ATL resection in non‐human primates. These investigations are best known for the profound post‐operative changes in social behaviour, including emotional blunting and hypersexuality. However, Klüver and Bucy's primary aims were to establish whether these bilateral lesions led to high‐level perceptual deficits, namely visual and auditory associative agnosias or, as referred to by these authors, ‘psychic blindness’. Indeed, this set of studies detail a broad symptom complex that was chiefly characterised by a failure to generate the meaning of visual and auditory stimuli. Therefore, it appears that their subjects were exhibiting multimodal semantic deficits that might explain, and not just co‐present with, the social‐affective disturbances.

In more recent years, the social neurosciences have seen another rise in interest regarding the specific role played by the ATL (for a review, see Olson et al., 2013). In particular, there emerged the *social knowledge hypothesis*, which states that this region supports a domain‐specific class of semantic knowledge: social concepts (Ross & Olson, 2010; Simmons et al., 2010; Zahn et al., 2007). Although this account acknowledges supporting evidence from within comparative and behavioural neurology, it is primarily based on functional neuroimaging data, which specifically points to the dorsolateral and polar ATL (also see Zahn et al., 2007).

Another long‐standing series of studies have implicated the ATL in more general forms of semantic processing (Lambon Ralph et al., 2017). These include detailed neuropsychological investigations of a disorder known as semantic dementia (SD). The SD syndrome falls within the spectrum of frontotemporal dementia and exhibits relatively focal atrophy and hypometabolism centred on the bilateral ATLs (Mummery et al., 2000; Nestor et al., 2006). This is coupled with a progressive, central impairment of semantic memory that is evident in both expressive and receptive semantic tasks, and across all modalities including spoken and written language, object use, picture‐based tasks, environmental sound tasks, and in olfaction and taste (Hodges & Patterson, 2007; Luzzi et al., 2007; Patterson et al., 2007; Piwnica Worms et al., 2010). Moreover, this human disorder displays striking parallels to the observations of Klüver and Bucy, in that the multimodal semantic deficit is accompanied by a range of socio‐affective deficits, which include impaired emotion recognition and empathy, impaired capacity for ToM, and a loss of person‐specific knowledge (Binney, Henry, et al., 2016; Ding et al., 2020; Edwards‐Lee et al., 1997; Snowden et al., 2018). This patient evidence is bolstered by a now extensive set of multi‐method studies that used electrophysiological recordings, neurostimulation techniques (TMS/tDCS) and/or functional neuroimaging in neurotypical samples (Binney et al., 2010; Binney & Lambon Ralph, 2015; Chan et al., 2011; Marinkovic et al., 2003; Pobric et al., 2008; Shimotake et al., 2015) all of which point to a role of the ATL in general semantic processing. However, as compared to the social knowledge hypothesis, this literature has converged upon a different subregion, the ventrolateral ATL, as the critical substrate for semantic knowledge representation. This includes the findings of ATL‐optimised fMRI studies and the data from SD which reveals that the ventrolateral ATL is, alongside the temporopolar regions, the most atrophied ATL subregion in this disorder (Binney et al., 2010; Galton et al., 2001; Mion et al., 2010). Moreover, it is noteworthy that Klüver and Bucy (1937) also remarked that the symptoms they observed in non‐human primate's failed to appear after resections limited to the dorsolateral convolutions of the temporal lobe. Nor did they present after severing connections of the temporal lobe to the frontal or to the occipital lobes. In other words, their observations highlighted a particular importance of the ventrolateral aspect of ATL in social‐affective behaviour.

The findings of the present study are most compatible with this second set of observations and implicate the ventrolateral ATL in both social and general semantic processing. To our knowledge, they represent the first firm demonstration using fMRI of ventrolateral ATL activation during the types of social (and more specifically, ToM) paradigms that are typically employed in the social neuroscience literature. This ATL subregion is frequently missing from fMRI studies probing ToM because of methodological considerations we were able to overcome (see below). The fact that three very different ToM paradigms evoked ventrolateral ATL activation suggest that it is a feature of ToM irrespective of the paradigm with which it is probed and therefore that it reflects a core cognitive component of ToM. Moreover, the fact that this activation overlapped directly with that evoked by a set of non‐social semantic judgements is consistent with the claim that engagement of the ATL by social tasks reflects access to a general class of domain‐general conceptual representations (Binney & Ramsey, 2020). The overlap we were able to reveal directly, using the present data, was only moderate in extent (although this will be dependent, in part, on thresholding, power and other methodological factors), but indirect comparison with the results of a wider set of prior studies (e.g., see Binney et al., 2010; Rice, Hoffman, et al. 2015; Visser & Lambon Ralph, 2011) also confirm that our ToM data implicate the same ventrolateral region engaged by general semantic processing.

Our results complement recent studies that found evidence of a role of the left ventrolateral ATL in accessing abstract social concepts (Binney, Hoffman et al., 2016 ; Rice et al., 2018) as well as other forms of social conceptual knowledge such as person semantics (Rice et al., 2018). Moreover, this region responds to meaningful stimuli across number of domains including faces, bodies, objects, and linguistic stimuli (Avidan et al., 2014; Collins & Olson, 2014; Harry et al., 2016; Ramot et al., 2019; Visser et al., 2012). This, alongside the fact that we were able to directly demonstrate ventrolateral ATL activation common to both nonverbal (the interacting shapes task) and verbal (the false belief vignettes) ToM tasks is consistent with the notion that the ventrolateral ATL is a supramodal hub engaged in semantic retrieval irrespective of the sensory, motor or linguistic modality through which concepts are probed (Lambon Ralph et al., 2017). Some prior fMRI studies have specifically pointed to the anterior fusiform gyrus as the supramodal centerpoint of the graded ATL semantic hub (Binney et al., 2010; Binney, Hoffman et al., 2016; Mion et al., 2010), whereas others have further implicated the ITG (e.g. Jackson et al., 2015; Rice et al., 2018; Visser et al., 2012). The present data suggest that it is the ITG, rather than the fusiform gyrus, that is particularly important for ToM, although the reasons for this are beyond the scope of the present data (also see below).

The dorsolateral ATL subregion previously implicated in the representation of social conceptual knowledge (e.g., Zahn et al., 2007) was notably absent within our main set of contrasts. This was somewhat unexpected and possible reasons follow. First, though, it is important to note that an absence of evidence for activation within this and other ATL subregions does not necessarily preclude the possibility that they also have a role in ToM and/or general semantic processing. Instead, it is possible that at the level of stringent thresholding that we employed in this study we have only been able to identify the strongest peaks of activation, or functional epicentres. In this case, it might be that activation in other ATL subregions only becomes detectable at a supra‐threshold level when there is increased power from higher levels of sampling or when more finely tuned contrasts are used to evoke greater effects sizes. If this were correct in the case of the dorsolateral ATL it would be consistent with claims that this subregion is less critical for semantic cognition (ergo ToM) than other subregions and, namely, the ventrolateral region (Binney, Hoffman et al., ^2016^). Indeed, we have contrasted semantic judgements made on social and non‐social stimuli in two prior studies and these revealed a sensitivity of activation to social stimuli in the dorsolateral ATL (Binney, Hoffman et al., 2016; Rice et al., 2018). However, these findings do not support a dual hub account of the ATL in which there are discrete functional subdivisions that support different classes of concept. Instead, they were in alignment with a ‘graded hub’ account in which the whole ATL comprises a single semantic hub, but has graded subspecialisations for processing certain types of conceptual information or ‘semantic features’ (Binney et al., 2012; Plaut, 2002; Rice, Hoffman, et al., 2015). This is because the adjacent ventrolateral ATL responded equally to both the social and non‐social stimuli and to a much greater extent than the dorsolateral subregion. According to graded hub hypothesis, the ventrolateral ATL region is the centre‐point of the hub and has a modality/domain/category‐general semantic function. The sensitivity of the dorsolateral/polar ATL to social stimuli may follow from this subregion's close proximity to and strong connectivity with the limbic system (via the uncinate fasciculus; Binney et al., 2012; Papinutto et al., 2016; Bajada et al., 2017), and could reflect a specialisation in the assimilation of, for example, emotion‐related or interoceptive information into coherent semantic representations (Olson et al., 2007; Rice, Hoffman, et al., 2015; Vigliocco et al., 2014).

A clear difference the way in which the ATL was engaged by the semantic judgements and the ToM tasks is that the latter appears far more bilateral. Moreover, ToM elicited bilateral ATL activation regardless of the verbal/non‐verbal nature of the stimuli. The role of the ATL in semantic cognition is proposed to be bilateral although, again, perhaps with graded specialisations towards processing verbal semantic information in the left hemisphere (Lambon Ralph et al., 2001; Rice, Hoffman, et al., 2015). The role of the ATL in social cognition has been ascribed with a right lateralisation within some accounts that draw mainly on neuropsychological patient evidence (Borghesani et al., 2019; Gainotti, 2015; Gorno‐tempini et al., 2003; Irish et al., 2014; Rice et al., 2017; Zahn et al., 2009), although the fMRI studies reviewed above (Binney, Hoffman et al., 2016; Rice et al., 2018; Ross & Olson, 2010) indicate bilateral involvement (also see Arioli et al., 2020; Catricalà et al., 2020; Lin et al., 2018; Pobric et al., 2016; Rice, Lambon Ralph, et al., 2015). Contrary to all these prior findings, the present ROI results are suggestive of a greater response to ToM tasks in the left as compared to the right ATL. Within the left hemisphere, there was also an apparent medial‐lateral differentiation in the responses of the ATL to the semantic task and the ToM task. A medial‐leaning profile of semantic activation during the picture version of the CCT task has also been reported by Visser and Lambon Ralph (2011) and Rice, Hoffman, et al. (2015) also found greater medial ATL activation for nonverbal relative to verbal semantic tasks. Initially, these observations could appear consistent with some variation of a dual hub hypothesis. That is, if it were not for task overlap in the ITG. Indeed, they are better accounted for by a graded semantic hub account in which there is a supramodal (ergo domain‐invariant) centre point that is flanked by a medial‐to‐lateral gradient of specialisation of semantic function that manifests differential (but not selective) responses to certain types of concepts (Binney et al., 2012; Lambon Ralph et al., 2017); more medial ATL regions show greater responsiveness to picture‐based materials than other types of material and, therefore, concrete concepts (Clarke & Tyler, 2015; Hoffman et al., 2015; Visser et al., 2012), and more lateral regions exhibit the opposite pattern, with greater activation for auditory stimuli and, therefore, abstract concepts that are more dependent on language experience (Hoffman et al., 2015; Scott et al., 2000; Visser & Lambon Ralph, 2011). The fact that the main ToM task activated the lateral ATL more than medial areas of the ATL is surprising when one considers the fact that it used visual stimuli. A possible explanation for this is that the meaning of the moving stimuli is not directly observable but instead must be inferred, and this may be both (a) mediated by the language system in the form of a running narrative, and (b) drawing on fairly abstract concepts (e.g., *cooperate*). Unfortunately, our data are not suitable for directly addressing this possibility, and the reasons for these differences remain unclear. Therefore, an interesting aim for future neuroimaging studies is to explore factors (e.g., task; stimulus modality) that could potentially drive differences in the activation of bilateral ATL subregions both in the context of social and general semantic tasks.

### 4.2 The status of the ATL in neurobiological accounts of social cognition

Animal ablation studies (Brown & Sharpey‐Schafer, 1888; Klüver & Bucy, 1937) and case descriptions of the profound consequences for humans of focal ATL lesions (Terzian & Dalle Ore, 1955) and degeneration (e.g., Edwards‐Lee et al., 1997) provided some relatively early clues as to the importance of the anterior temporal cortex for socio‐affective competences. Nonetheless, the ATL often does not feature prominently within contemporary neurobiological frameworks for understanding social behaviour (Adolphs, 2009; Decety & Lamm, 2007; Lieberman, 2007; Spunt & Adolphs, 2017; van Overwalle, 2009). It is overshadowed by prefrontal, medial and lateral temporoparietal regions and seemingly attributed with an ancillary status. This could be due, at least in part, to the predominance of fMRI in the social neurosciences and the fact that this technique is typically blind to activation in a significant proportion of this region (Devlin, 2002). Inconsistencies in the presence and location of ATL activation across various social domains, relative to the TPJ, for example, could explain a modest appetite for further exploring the region's contribution.

Here, and in two prior ATL‐optimised fMRI studies (Binney, Hoffman et al., 2016; Rice et al., 2018), we have shown that when steps are taken to alleviate the technical limitations of the fMRI technique, robust ATL activations are observed across a variety of social stimuli and social tasks. Activation also occurs in a ventrolateral ATL region that is one of the most affected in patients with both striking semantic and social impairments (Binney et al., 2010; Binney, Henry, et al., 2016; Kumfor et al., 2016). Moreover, in the present study, we have demonstrated that left ventrolateral ATL activation is at least as robust, in extent and magnitude, as that of another key social region (the TPJ), and at least as consistent across different tasks and stimuli. Overall, we interpret this as initial evidence from neurotypical samples to complement that obtained from patient studies, that the ventrolateral ATL is of equal functional import to social cognition as other key nodes of the ‘social brain’ (such as the TPJ, the mPFC and the precuneus).

Several authors have argued that progress in social neuroscience theory will rapidly accelerate if it embraces established and detailed models from within other more general domains of cognition (Amodio, 2019; Ramsey & Ward, 2020; Spunt & Adolphs, 2017). Taking a similar perspective, we have recently proposed that a unifying feature amongst many forms of social cognitive processing is the retrieval of conceptual knowledge, and that it could be productive to understand social cognition to essentially be an example of semantic cognition (Binney & Ramsey, 2020). This would appear a reasonable viewpoint given that social interaction is, at its core, a process of *meaningful* exchange between persons. The main practical implication of this proposal, at least for the present discussion, is that social and semantic cognition rely on the same cognitive and brain mechanisms and this positions the ventrolateral ATL at the heart of social cognition.

Beyond the ATL, our findings also confirm a role of the TPJ, the mPFC, and the precuneus in ToM (Frith & Frith, 2003; Frith & Frith, 2006; Jacoby et al., 2016; Saxe & Kanwisher, 2003; van Overwalle, 2009). However, the TPJ and mPFC are also activated by general semantic processing. There are also other areas of activation that overlap across the two tasks, including the pMTG and bilateral inferior occipito‐temporal regions. The overlap suggests these regions serve a domain‐general role rather than one that is specialised towards processing social information. While there is evidence for a selective role of the right TPJ and mPFC in social and moral processing (Saxe & Kanwisher, 2003; Saxe & Wexler, 2005; Young et al., 2010 ), there is also evidence that they are engaged by a wide range of tasks including those outside the social domain (Bzdok et al., 2016; Cabeza et al., 2012; Diveica et al., 2021,; Humphreys et al., 2020; Seghier et al., 2010; van Overwalle, 2009). In other recent work, we have considered the possibility that activation of frontal regions, the TPJ and pMTG during ToM tasks, also reflects engagement of domain‐general processes related to semantic cognition (Binney & Ramsey, 2020). However, a key distinction between this set of hypotheses and those centred on the ATL is that, rather than be considered additional components of a semantic representational system, these regions are purported to be involved in cognitive control processes (Diveica et al., 2021 ). A fuller discussion of these issues is beyond the scope of the present article, but there is a growing need to re‐evaluate the relative contribution of all these regions, as well as develop a better understanding of the way they interact in service of social cognition.

### 4.3 Conclusions and future directions

In conclusion, our findings support the claim that the ventrolateral ATL is an important contributor to social cognition and point to a specific role as a domain‐general hub for conceptual knowledge representations that help inform our understanding of others and guide our own meaning‐driven social behaviours. A key methodological determinant underpinning these findings was the use of a neuroimaging technique that maximises the signal obtained from across the entire ATL region. However, the present study is also limited by its methodology. To a large extent, fMRI remains the predominant mode of investigation in the social neurosciences. However, it cannot be escaped that the inferences it allows are merely correlational and not at all causal. For this reason, the field needs to increasingly turn to patient models such as stroke, temporal lobe epilepsy, and frontotemporal dementia (Kumfor, Hazelton, et al., 2017; Kumfor, Honan, et al., 2017; Rankin, 2020, 2021), as well as non‐invasive techniques, such as transcranial magnetic stimulation, that can be used to more directly probe the neural architecture of cognition in neurological healthy samples. This will enable us to get a firmer grasp on key questions including those regarding the laterality of function within the ATL and the TPJ, as well as the functional necessity of distinct subregions.

## CONFLICT OF INTEREST

The authors declare no potential conflicts of interest.

## Supporting information


**Appendix S1** Supporting information

## Data Availability

Following open science initiatives (Munafò et al., 2017), behavioural and neuroimaging data are openly available on the Open Science Framework project page (https://osf.io/v2gt5/).
